# A multicenter, open-label study for efficacy and safety evaluation of anagrelide in patients with treatment-naïve, high-risk essential thrombocythemia as a primary treatment

**DOI:** 10.3389/fonc.2022.989984

**Published:** 2022-11-23

**Authors:** Ja Min Byun, Ho Young Kim, Seung-Hyun Nam, Ho-Jin Shin, Seulki Song, Jinny Park, Sang Hoon Han, Yong Park, Young Jin Yuh, Yeung-Chul Mun, Young Rok Do, Sang Kyun Sohn, Sung Hwa Bae, Dong-Yeop Shin, Sung-Soo Yoon

**Affiliations:** ^1^ Department of Internal Medicine, Seoul National University Hospital, Seoul, South Korea; ^2^ Department of Internal Medicine, Hallym University Medical Center, Anyang, South Korea; ^3^ Department of Internal Medicine, Kyung Hee University at Gangdong, Seoul, South Korea; ^4^ Division of Haematology-Oncology, Department of Internal Medicine, School of Medicine, Medical Research Institute, Pusan National University Hospital, Busan, South Korea; ^5^ Cancer Research Institute, Seoul National University Hospital, Seoul, South Korea; ^6^ Division of Hematology, Department of Internal Medicine, Gachon University Gil Medical Center, Incheon, South Korea; ^7^ Department of Internal Medicine, Jeju National University Hospital, Jeju National University School of Medicine, Jeju, South Korea; ^8^ Division of Hemato-Oncology, Department of internal medicine, Korea University School of Medicine, Seoul, South Korea; ^9^ Department of Internal Medicine, Inje University College of Medicine, Sanggye Paik Hospital, Seoul, South Korea; ^10^ Department of Internal Medicine, Ewha Womans University Mokdong Hospital, Ewha Womans University College of Medicine, Seoul, South Korea; ^11^ Department of Internal Medicine, Keimyung University Dongsan Hospital, Keimyung University School of Medicine, Daegu, South Korea; ^12^ Department of Internal Medicine, Kyungpook National University Hospital, School of Medicine, Kyungpook National University, Daegu, South Korea; ^13^ Department of Internal Medicine, Daegu Catholic University School of Medicine, Daegu, South Korea

**Keywords:** essential thrombocythemia, high risk, Anagrelide, phase IV clinical trial, myeloproliferative neoplasms

## Abstract

As the discussion of first-line anagrelide treatment is ongoing, we aimed to prospectively examine the efficacy and safety of anagrelide in cytoreduction therapy-naïve high risk essential thrombocythemia (ET) patients in Korea. Seventy patients from 12 centers were treated with anagrelide monotherapy for up to 8 weeks, followed up until 24 months. At week 8, 50.0% of the patients were able to achieve platelet < 600 x 10^9^/L, and by 12 months, 55/70 (78.6%) patients stayed on anagrelide, and 40.0% patients showed platelet normalization. 14 patients required additional hydroxyurea (HU) for cytoreduction. The median daily dose of needed HU was 500mg (range 250mg – 1500mg). The efficacy was independent of the somatic mutation status. There were 4 thromboembolic events and 7 bleeding events during the follow-up period. The most common adverse events associated with anagrelide use were headache, followed by palpitation/chest discomfort, edema and generalized weakness/fatigue. 7 patients wished to discontinue anagrelide treatment due to adverse events (3 due to headache; 2 due to edema; 1 due to palpitation and 1 due to skin eruption). All in all, first-line anagrelide treatment showed a favorable response with tolerable safety profiles regardless of somatic mutation status.

## Introduction

Essential thrombocythemia (ET) is a type of myeloproliferative neoplasm characterized by abnormal proliferation of megakaryocytes in the bone marrow leading to elevated platelet counts (1). Since ET patients have an almost normal survival, any survival effect of treatment is very difficult to prove. Thus, the goal of ET treatment is reduction of thrombotic and hemorrhagic complications, and platelet reduction to <400 x10 ^9^/L. While cytoreductive therapy with or without low-dose aspirin is the mainstay of thrombosis risk reduction, the optimal choice of a therapeutic agent is less clear. Several agents, including hydroxyurea, anagrelide, and interferon are used for this purpose, but there are only a handful of data directly comparing these agents (2–7). As such, there is a gap in preferred therapeutic agents among the continents: based on the ANAHYDRET study which showed non-inferiority of anagrelide to hydroxyurea (5), anagrelide is used as first-line therapy for high-risk ET patients in Korea. On the other hand, due to concerns about leukemogenesis observed in the EXELS study (6, 8, 9) and conflicting results of PT-1 trial data (7), anagrelide remains a second-line therapy in Europe.

Anagrelide is an amidazoquinazolin derivative, originally developed as an anticoagulation drug, which was shown to have a potent platelet reducing effect (10). It is the only platelet-specific cytoreductive drug known, having no inhibitory effects on red or white cell progenitor proliferation. It reduces platelet production by inhibiting megakaryocyte colony development, thus producing a left-shift in megakaryocyte maturation, reducing megakaryocyte size, and maturation (11). As discussion of anagrelide efficacy and safety is ongoing and a consensus has not been reached, we aimed to prospectively examine the efficacy and safety of anagrelide as cytoreduction therapy-naïve high-risk ET patients in Korea.

## Methods

### Design overview

This was a multi-center, prospective observation study (ClinicalTrials.gov identifier: NCT03232177). The aim of the study was to examine the efficacy and safety of first-line anagrelide treatment in high-risk Korean ET patients. The primary objective was to determine the percentage of patients who had hematologic response, defined as platelet count < 600 x 10 ^9^/L by week 8 on anagrelide monotherapy. The secondary objectives included (1) platelet normalization rates (platelet < 400 x 10^9^/L) at week 8 and 12 months per European LeukemiaNet guidelines (12) (2); platelet reduction by more than 50% at week 8 and 12 months; (3) anagrelide safety, tolerability and compliance at 12 months. We also evaluated somatic mutation profiles and anagrelide efficacy as an exploratory objective. The interest in mutation profiling was instigated by 2 recent small studies suggesting that anagrelide may be more effective in *JAK2* positive patients than *CALR* positive patients (13, 14). Specifics target sequencing methods are available in **Supplementary Materials**.

### Study population

Patients older than 18 years with ET diagnosed according to 2016 WHO classification (1) were screened. Those participants with high-risk ET [age older than 60 years or a history of vascular complications (15–17)] and cytoreductive treatment naïve were invited to participate in the study, regardless of mutational status. Patients with underlying medical conditions requiring active interventions, inadequate organ (cardiac, hepatic, renal, pancreatic) functions, pregnancy, concurrent malignancies, or taking phosphodiesterase III/IV drugs were excluded. The study was conducted according to the Declaration of Helsinki and was approved by the institutional review board (IRB) of each hospital. Informed consent was taken from all patients before participating in any study-related procedure.

### Interventions

From week 1 to week 8, anagrelide monotherapy was required. Patients were started on anagrelide 0.5mg twice a day and after 1-week dose escalation was allowed. From week 9, additional agents for cytoreduction were allowed per attending physician’s discretion. The maximum anagrelide dose allowed was 10mg/day (2.5mg four times a day). Patients were followed up at week 1, week 4, week 8, 3 months, 6 months, 9 months, 12 months, 18 months and 24 months for lab testing and drug compliance monitoring. The adverse events (AE) were assessed according to the National Cancer Institute Common Terminology Criteria for Adverse Events version 4.03 (available at: https://evs.nci.nih.gov/ftp1/CTCAE/CTCAE_4.03/CTCAE_4.03_2010-06-14_QuickReference_8.5x11.pdf). The bleeding and thrombotic complications were graded according to International Society on Thrombosis and Hemostasis (ISTH) (18), which are presented in **Supplementary Materials** in details.

### Statistical analysis

Fisher’s exact test was used for nominal variables, and Mann-Whitney U test was used for continuous variables. For all statistical analyses of effective variables, two-tailed tests were performed. *p-*values of <0.05 were considered statistically significant. All data were analyzed using the Statistical Package for the Social Sciences software (IBM^®^ SPSS^®^Statistics, version 25.0).

## Results

### Baseline characteristics

**Table 1** shows the baseline characteristics of all 70 patients (34 males and 36 females) enrolled. The median age at ET diagnosis was 69 years (range 24 – 90), and time to study enrollment from diagnosis was median 13 days. The majority of the patients harbored *JAK2V617F* mutation (48/69, 68.6%) and there were 13 (18.8%) triple-negative patients. As for the adjunct anticoagulation therapy, 51/70 (72.9%) took aspirin, 14/70 (20.0%) plavix, 2/70 (2.9%) enoxaparin, 2/70 (2.9%) edoxaban and 1/70 (1.4%) dabigatran.

**Table 1:** Baseline characteristics.

Characteristics	Total (N=70)
**Sex, male (N, %)**	34
**Age at study enrollment, years (median, range)**	69 (24-90)
>60 years old	50 (71.4)
**Diagnosis to study enrollment, days (median, range)**	13 (0-792)
**Mutation status (N, %)**	
*JAK2V617F*	48/69 (68.6)
*CALR*	6/69 (8.6)
*MPL*	6/69 (8.6)
Triple negative	13/69 (18.8)
**Previous drug exposure (N, %)**	
Hydroxyurea	0 (0)
Anagrelide	0 (0)
Interferon	0 (0)
**Baseline laboratory findings (mean, ± SD)**	
WBC (x10^9^/L)	10.4 (0.4)
Hemoglobin (g/dL)	13.8 (0.2)
Platelet (x10^9^/L)	931.4 (40.0)
**Baseline platelet > 1000 x 10^9^/L**	21 (30.0)
**Cardiovascular risk factors (N, %)**	
Hypertension	29 (41.4)
Diabetes	11 (15.7)
Dyslipidemia	10 (14.3)
BMI > 25	27 (38.6)
**Prior history of thrombosis**	13 (18.6)
**IPSET-Thrombosis risk stratification**	
Low	7 (10.0)
Intermediate	6 (8.6)
High	57 (81.4)

ET, essential thrombocythemia; SD, standard deviation; WBC, white blood cell count; BMI, body mass index; IPSET, International Prognostic Score.

### Treatment outcomes

The response was evaluated in the 64 patients who stayed on anagrelide treatment at week 8 (**Table 2**). Platelet normalization was documented in 20.3% of the patients by week 8, and 40.0% by month 12. Platelet reduction by more than 50% was seen in 18.8% of the patients by week 8, and in 43.6% of the patients by month 12.

**Table 2:** Treatment related outcomes.

Parameters	N, %
Response*	
Platelet < 600 (x10^9^/L) at 8 weeks	32/64 (50.0)
Platelet reduction ≥ 50% at 8 weeks	12/64 (18.8)
Platelet normalization (≤ 400 x 10^9^/L) at 8 weeks	13/64 (20.3)
Platelet reduction ≥ 50% at 12 months	24/55 (43.6)
Platelet normalization (≤ 400 x 10^9^/L) at 12 months	22/55 (40.0)
**Additional cytoreductive agents use**	
Hydroxyurea	14/69 (20.3)
*250mg*	3
*500mg*	8
*1000mg*	1
*1500mg*	2
Thromboembolic events, patients**	
Cerebral infarction (major arterial thrombosis)	3
Angina pectoris (minor arterial event)	1
Bleeding events, patients*	
GI bleeding (major bleeding event)	1
Ecchymosis (minor bleeding event)	2
Epistaxis (minor bleeding event)	3
Intracranial hemorrhage (major bleeding event)	1

*Response was calculated from patients stayed on anagrelide treatment regardless of compliance.

**According to predefined criteria (19).

As shown in **Table 2**, there were 4 thromboembolic events (20) (3 major arterial thromboses; 1 minor arterial event) and 7 bleeding events (2 major bleeding events; 5 minor bleeding events) during the follow-up period. There were no venous thrombosis/events during the follow-up. One patient with a history of carotid artery stenosis and hepatitis B suffered from an angina pectoris attack requiring intervention. Of the 3 patients who experienced cerebral infarction, one had the previous history of transient ischemic attack, one had a history of thyroid cancer, and one had alcoholic liver cirrhosis. As for the bleeding events, the patient who had intracranial hemorrhage had a history of cerebral infarction and hypertension.

### Adverse events

As for tolerability, 64/70 (91.4%) patients remained on anagrelide monotherapy by week 8. Among the 64 patients remaining on anagrelide, all but 1 had taken ≥80% of the prescribed medication. By 12 months, 55/70 (78.6%) patients stayed on anagrelide and 54/55 patients showed good compliance to the drug (**Table 3**).

**Table 3:** Adverse events.

Parameters	N
**Stayed on anagrelide treatment**	
Baseline	70/70 (100)
Week 1	69/70 (98.6)
Week 4	66/70 (94.3)
Week 8	64/70 (91.4)
6 months	59/70 (84.3)
12 months	55/70 (78.6)
24 months	52/70 (74.3)
Medication compliance*	
Week 1	65/69 (94.2)
Week 4	63/66 (95.5)
Week 8	63/64 (98.4)
12 months	54/55 (98.1)
**Reasons for anagrelide discontinuation**	
Adverse events	7/17 (41.2)
Lost to follow-up	3/17 (17.6)
Consent withdrawal	7/17 (41.2)
**Symptoms related to anagrelide, any (grade ≥3), events**	
Headache	26 (4)
Palpitation/chest discomfort	19 (2)
Edem	17 (4)
Generalized weakness/fatigue	17 (1)
Dyspepsia	12(4)
Itching/pruritis	10(3)
Diarrhea/loose stool	10 (2)
Dizziness/light headedness	10 (0)
Epigastric pain	8 (5)
Dyspnea	7 (1)
Abdominal pain	3 (0)
Skin eruption/rash	5 (1)
Bone pain	5 (1)
Anorexia	5 (2)
Constipation	4 (1)
Neuropathy/tingling sensation	4 (2)
Nausea	3 (1)
Uncontrolled blood pressure	3 (1)
Myalgia	3 (0)
Liver function test elevation	2 (1)
Creatinine elevation	2 (2)
Insomnia	1 (1)
Serum glucose elevation	1 (0)

*Taken ≥80% of the prescribed drugs.

**Figure 1:**
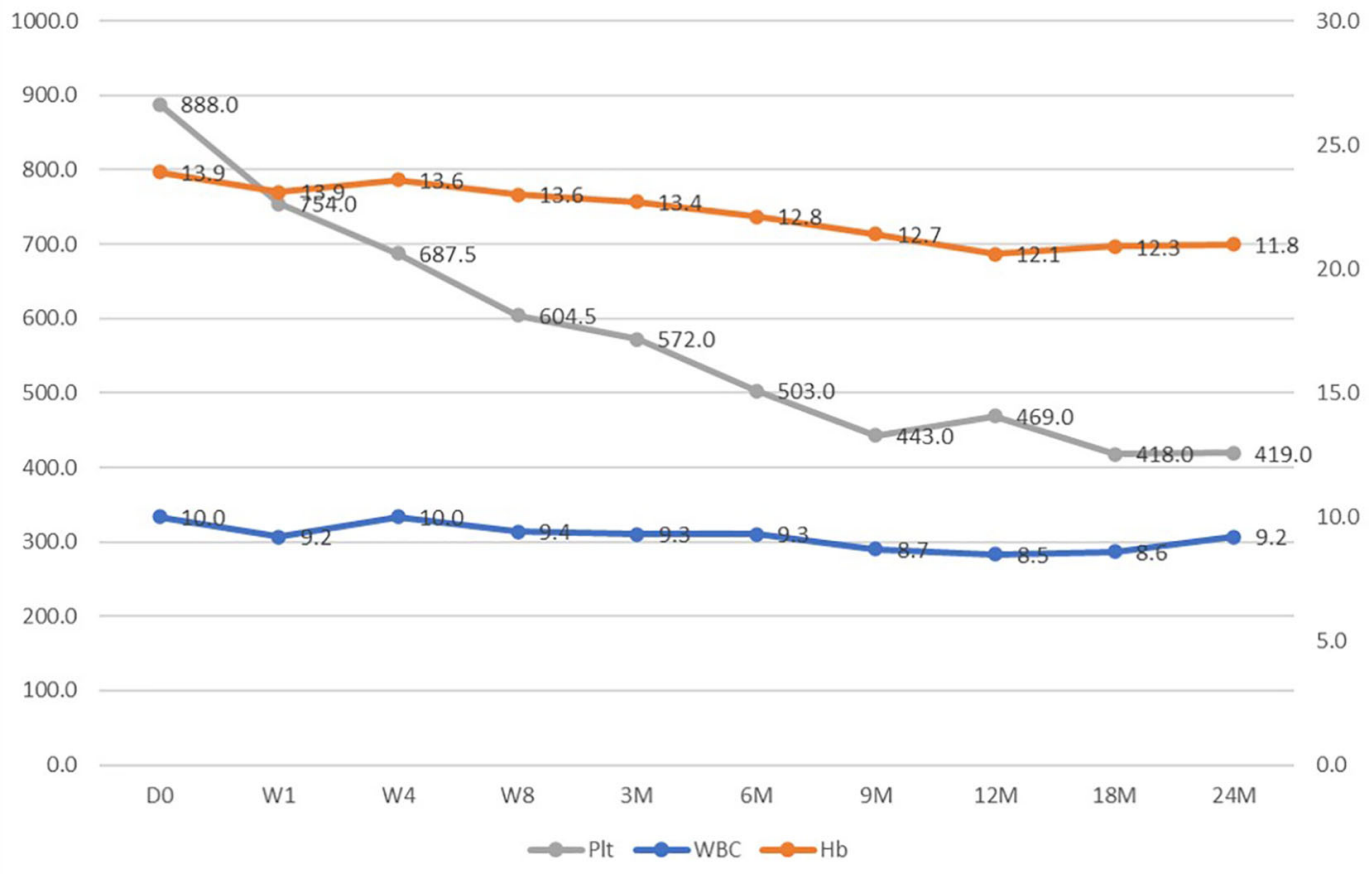
Hemogram changes over the treatment course.

**Figure 2:**
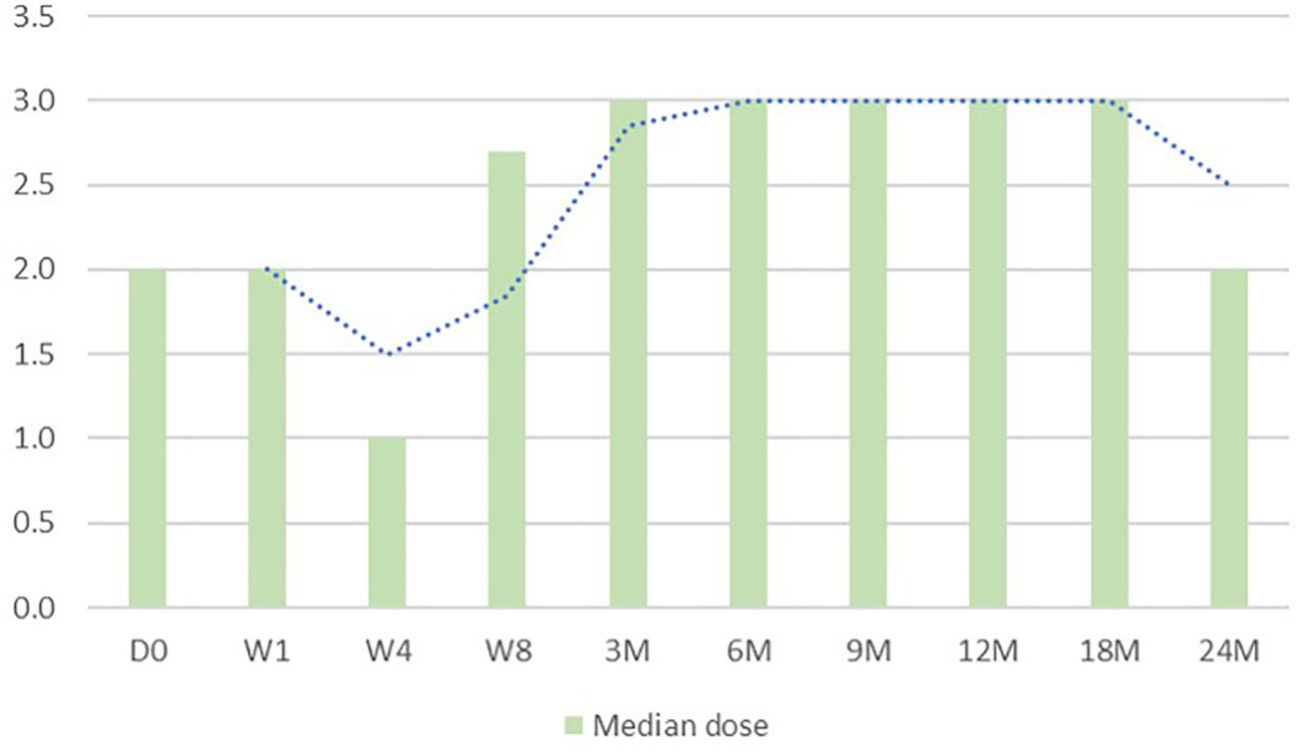
Changes in anagrelide dose over the treatment course.

Anagrelide treatment had minimal effects on white blood cell counts and hemoglobin level as shown in **Figure 1**. The median dose of anagrelide required was 2.5mg per day (**Figure 2**). Fourteen patients required additional hydroxyurea (HU) for cytoreduction. The median dose of needed HU was 500mg (range, 250mg – 1500mg). Fortunately, there were no differences in adverse events profile among those taking additional hydroxyurea versus those who did not.

The most frequent AE associated with anagrelide use was headache, followed by palpitation/chest discomfort, edema and generalized weakness/fatigue. The most common AE ≥ grade 3 was epigastric pain, followed by headache, edema and dyspepsia. 7 patients wished to discontinue anagrelide treatment due to adverse events (3 due to headache; 2 due to edema; 1 due to palpitation and 1 due to skin eruption). There were no acute leukemia transformation or myelofibrosis transformation during the follow-up.

### Somatic mutational profile

**Figure 3:**
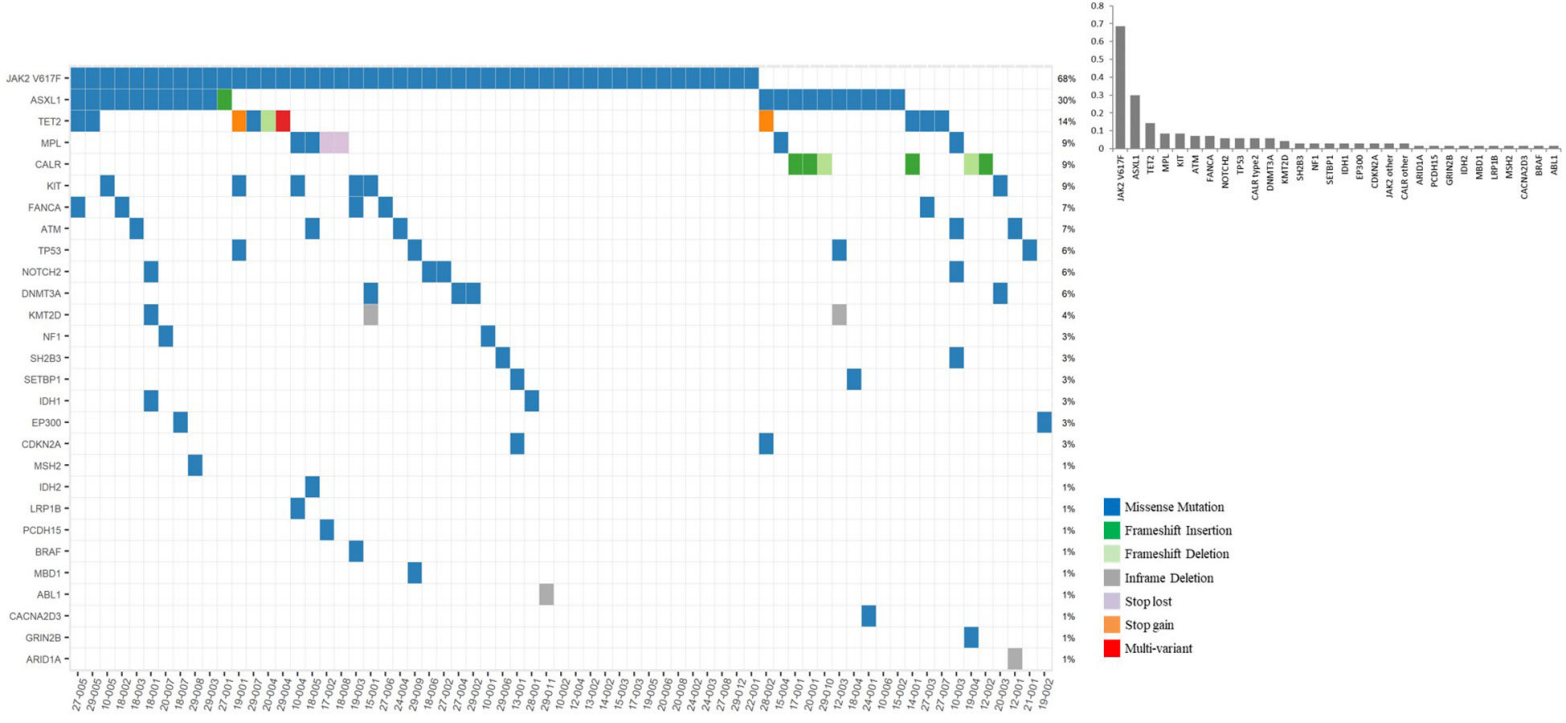
Baseline somatic mutation profiles of essential thrombocythemia patients treated with anagrelide.

The overall somatic mutational profile is shown in **Figure 3**. Interestingly, 4 patients showed concurrent *JAK2V617F* mutation and *MPL* mutation. There were no patients harboring *JAK2 exon 12* mutation. Other than the known driver mutations, *ASXL1* was identified in 21/70 (30.0%) patients, followed by *TET2* 10/70 (9.8%) and *KIT* 6/70 (8.6%).

**Figure 4:**
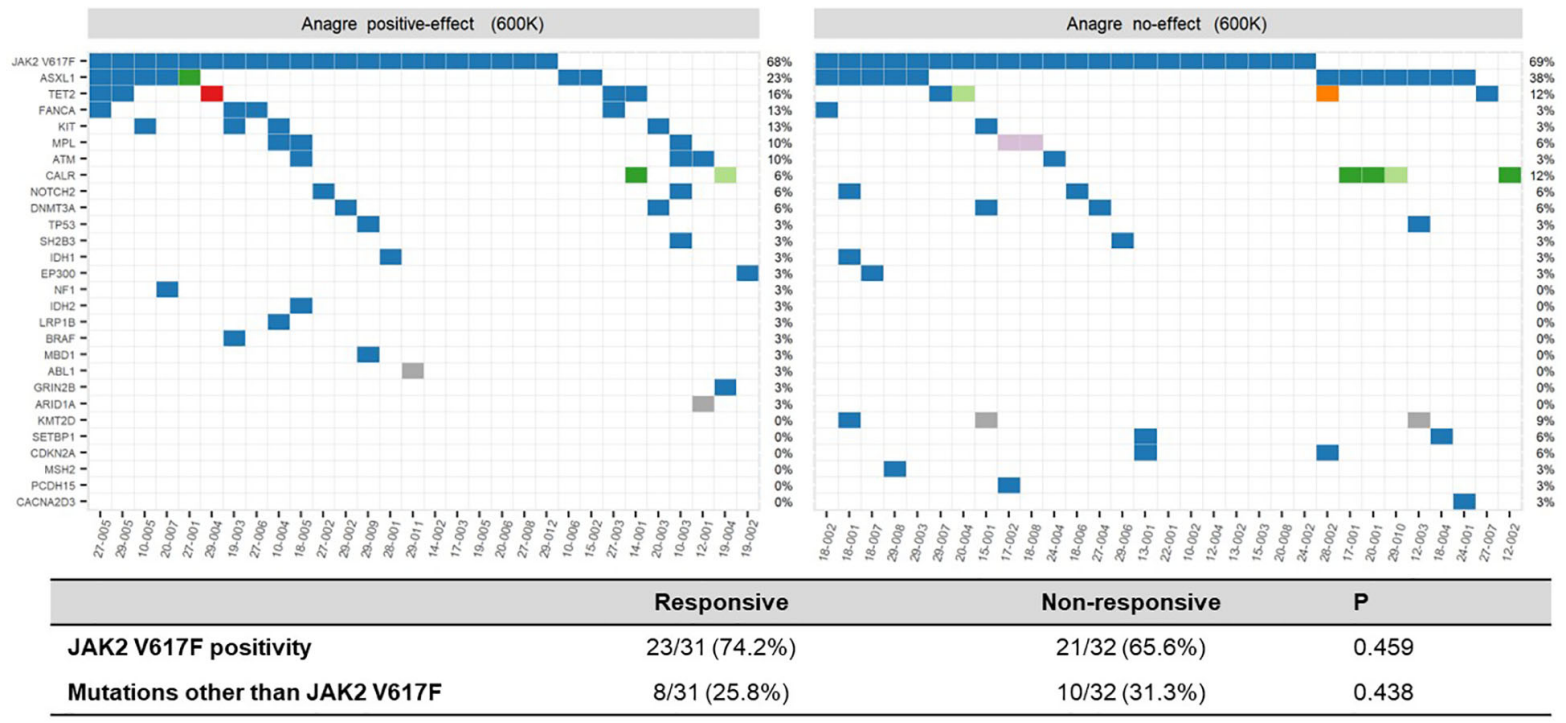
Somatic mutation landscape according to treatment efficacy, patients reaching platelet level < 600 x 10^9^/L versus ≥600 x 10^9^/L at week 8.

**Figure 4** shows the mutation status according to treatment efficacy. The mutation status was not related to anagrelide treatment response. More specifically, among patients meeting primary endpoint of platelet count < 600 x 10^9^/L by week 8 74.2% harbored *JAK2V617F* mutation while 65.6% of the non-responders harbored *JAK2V617F* mutation (p=0.459). Non-*JAK2V617F* mutations were noted in 8 responders (25.8%), while 10 (31.3%) non-responders harbored mutations other than *JAK2V617F* (p=0.438).

## Discussion

In this study, we present the efficacy and safety results of first-line anagrelide treatment for high-risk ET based on prospective clinical data. The importance of our study lies in that 1) this is a rare prospective study using anagrelide as first-line; 2) the efficacy of anagrelide monotherapy was tested, and in relation to genetic background; 3) ethnically homogeneous population with high-risk ET were enrolled. Overall, we report a 50% response rate with anagrelide monotherapy and a 40% complete remission rate at 1 year with 78.6% of the patients staying on the medication with good tolerability.

It is known that approximately 60% of ET patients harbor *JAK2V617F* mutation (21). Likewise, 68.6% (48/69) of our patients showed *JAK2V617F* mutation (**Figure 1**). Although relatively small in number, along with a previous Japanese study reporting JAK2V617F mutation rate at 64.2% for ET (22), there seem to be no ethnical differences in ET mutational profiles. Interestingly, there were only 6 patients (8.6%) harboring *CALR* mutation. This is probably because our study enrolled only high-risk ET patients defined as older than 60 years old or having a history of vascular complications. It is well-known that *CALR*-mutated ET patients show higher platelet count and lower thrombotic risk compared to *JAK2V617F*-positive ET patients (23).

Interestingly, there were 4 patients who harbored both *JAK2V617F* and *MPL* mutations. *MPL* mutations were noncanonical mutation (*T374A*) in 3 patients, but a canonical mutation in the other one patient (*W515L*). Somatic mutations in *TP53*, an important prognostic factor in MPN, was found in 4 patients (5.8%). Another DNA repair gene, *ATM* mutation was found in 5/69 (7.2%) patients. Longer follow-ups are required to determine the prognostic values of these mutations.

The efficacy of anagrelide, on the other hand, was not determined by mutational status. Half of the enrolled patients achieved platelet level < 600 x 10 ^9^/L by week 8 with anagrelide monotherapy, irrespective of driver mutation status (**Table 2**; **Figure 4**; **Supplementary Figure**). This is numerically lower than the one reported by Ito et al. (22), who reported response rate of 83%. We believe such discrepancy is due to the difference in study schema and study population. At 12 months, regardless of driver mutation status, 40.0% of the patients achieved platelet normalization and 43.6% of the patients saw more than 50% reduction in platelet level from baseline. Since anagrelide is not a specific targeted agent but rather its platelet-reducing effect is mediated through reduction in pro-platelet formation (6, 8), it is understandable that the mutation status does not affect the drug’s efficacy.

The medication was relatively well tolerated: 74.3% of the patients stayed on anagrelide at 24 months with a median dose of 2 mg. Fortunately, no one experienced anemia development with anagrelide treatment. As with previous reports (3, 19, 22), the 2 most common adverse events were headaches and palpitations. In our study, 3 patients decided to discontinue anagrelide due to headache and 1 due to palpitation. Overall, there were only 4 thromboembolic events and 7 bleeding events during the 24-month follow-up period. Numerically, these are fewer compared to previous studies, which reported 11.5% (14/122) major events and 36.9% (45/122) minor events (5). Interpretation of such discrepancy warrants caution: traditionally Asians have been associated with lower incidence of thromboembolism (19, 24, 25) but recently it was reported that Korean patients have similar frequency of thrombosis compared to Western patients (26, 27). In fact, one of the major limitations of our study is that we are not able to determine if anagrelide can significantly decrease thrombosis or bleeding risk because this was a single-arm study without a control arm and because only 4 thrombotic events occurred during a follow-up time of 24 months. Since the fundamental goal of treatment in high-risk ET is a reduction of thrombosis and hemorrhage through platelet reduction to preferably normal range, we aim to investigate further if platelet response correlates with vascular events through a longer follow-up. Lastly, also due to short follow-up period, we were not able to document secondary transformation of ET. This, too, warrants a longer follow-up study.

In conclusion, our study supports the use of anagrelide as a first-line cytoreductive agent in high-risk ET. Longer and more detailed follow-up on thrombotic and bleeding complications should ensue.

## Data availability statement

The datasets presented in this study can be found in online repositories. The names of the repository/repositories and accession number(s) can be found below: https://www.ncbi.nlm.nih.gov/, PRJNA853096.

## Ethics statement

The studies involving human participants were reviewed and approved by Seoul National University Hospital. The patients/participants provided their written informed consent to participate in this study.

## Author contributions

Contribution: D-YS and S-SY created the concept and design; all authors provided the study materials or patients; JB and D-YS collected and assembled the data; and all authors contributed to the writing and the final approval of the manuscript.

## Funding

Anagrelide was provided by Yuhan Corporation. This work was partially supported by the National Research Foundation of Korea (NRF-2020R1F1A1076106).

## Conflict of interest

The authors declare that the research was conducted in the absence of any commercial or financial relationships that could be construed as a potential conflict of interest.

## Publisher’s note

All claims expressed in this article are solely those of the authors and do not necessarily represent those of their affiliated organizations, or those of the publisher, the editors and the reviewers. Any product that may be evaluated in this article, or claim that may be made by its manufacturer, is not guaranteed or endorsed by the publisher.
