# Supplementary material for: A multicenter, open-label study for efficacy and safety evaluation of anagrelide in patients with treatment-naïve, high-risk essential thrombocythemia as a primary treatment
Source: Front Oncol. 2022 Nov 23;12:989984. doi: 10.3389/fonc.2022.989984 (PMC9727180; doi:10.3389/fonc.2022.989984)
Supplement: Supplementary file 1 [file DataSheet_1.pdf]

Supplementary Materials

Methods -- Page 2-4

Supplementary Figure --- Page 5-8

References --- Page 9

Methods

Target sequencing

DNA from the patient's bone marrow and peripheral blood mononuclear cells were extracted using a QIAamp DNA mini genomic DNA kit (Qiagen, Hilden, Germany) according to the manufacturer's instructions. Then we constructed the exome capture libraries from native DNA using the Agilent SureSelect Target Enrichment protocol for Illumina paired-end sequencing library (Version C2, December 2018) and the SureSelectXT Custom probe (design ID: 3195611). Whole-exome sequencing was performed using the NovaSeq platform (Illumina, San Diego, USA). The paired-end sequences/reads preprocessing and variant calling were based on the in-house pipeline in accordance with Genome Analysis Toolkit (GATK)[1] best practices. Sequenced reads were aligned to the human reference genome (hg19) using the Burrows-Wheeler Alignment tool (BWA, version 0.7.10)[2] after trimming the Illumina adapter sequences and low quality reads using Trimmomatic-v0.39[3]. PCR duplicates from the sorted binary alignment map (BAM) were marked through Picard-v1.13's MarkDuplicate tool (https://broadinstitute.github.io/picard/). GATK IndelRealigner and BaseRecalibrator were employed for local realignment and base quality, respectively. Single nucleotide variants (SNVs) and Insertions and deletions (InDels) calling for each sample was performed with HaplotypeCaller [4]. The called variants in genomic variant call format (gVCF) were consolidated using the joint genotyping GenotypeGVCF followed by hard filtering of GATK. Furthermore, the low-quality variants (total or alternative read depth less than 10) and variant allele frequency (VAF) less than 5% were discarded. Then, we annotated variants in VCF with ANNOVAR [5] to perform filter-based functional annotation and Variant Effect Predictor (VEP)-release-96 [6] to explicate the gene-based information of canonical transcripts. Any

possible germline polymorphisms and ethnic-specific variants with a greater allele frequency of 0.1 in the normal population database called Genome Aggregation Database [7] (gnomAD_v2.1.1) were filtered. Finally, protein-coding variants except the synonymous variants with somatic mutational information in COSMIC database [8] were used for further analysis. Data generation was done in 69 out of the 70 patients due to sample quality issue.

Thromboembolic events

- Major arterial thrombosis: stroke, myocardial infarction, peripheral arterial disease, other arterial thrombosis.
- Major venous thrombosis: ileofemoral thrombosis, pulmonary embolism, splanchnic vein thrombosis, other major venous events.
- Minor arterial events: transitory ischemic attacks, angina pectoris, unstable angina, generalized convulsions, erythromelalgia, ocular symptoms, other peripheral arterial microcirculatory disturbances, other minor arterial events (eg, tinnitus, vertigo).
- Minor venous events: superficial thrombophlebitis, other minor venous events. Minor events were diagnosed based on patients' symptoms and clinical judgment of the investigator taking patient diary notes into account.

Bleeding events

- Major bleeding events: decrease in hemoglobin level >1 g/dL or red blood cell transfusion required.

- Minor bleeding events: no red blood cell transfusion required and decrease in hemoglobin level >1g/dL.

Supplementary Figure. Somatic mutation landscape according to treatment efficacy

(A) Platelet level reaching < 400 x 10^9/L vs ≥400 x 10^9/L at week 8

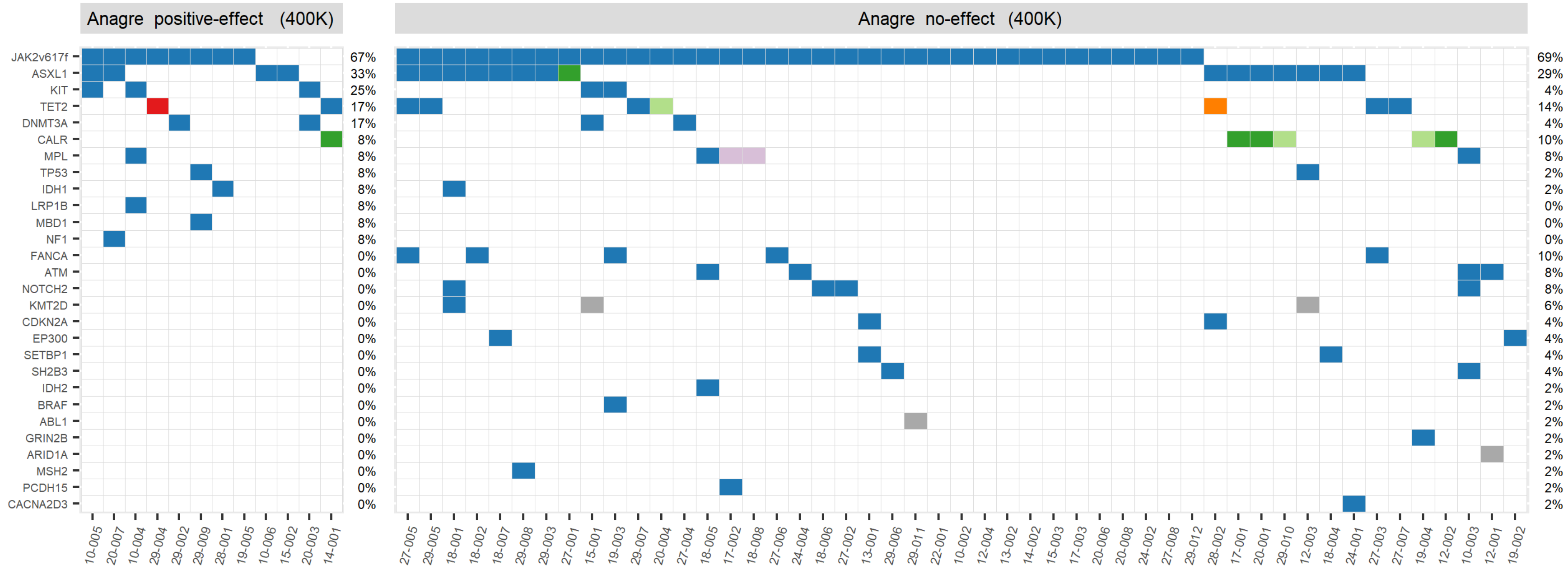

(B) Platelet dropping by 50% vs not at week 8

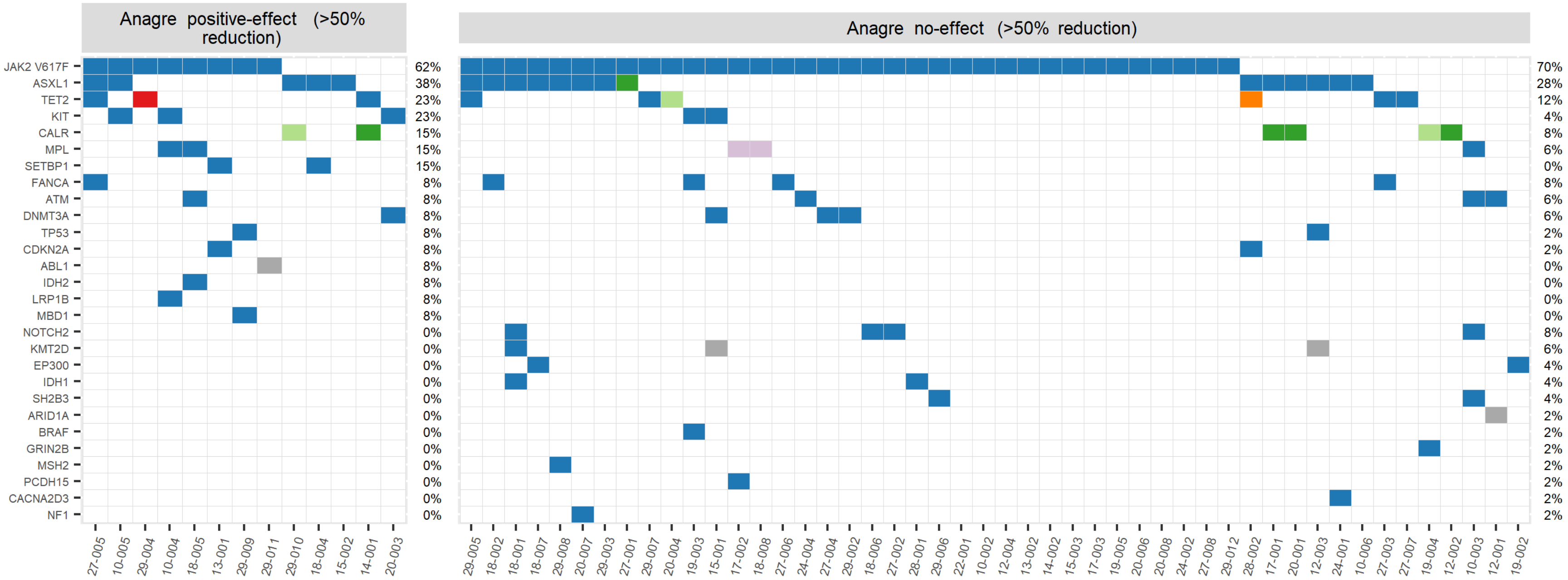

(C) Platelet level reaching < 400 x 10^9/L vs ≥400 x 10^9/L at 12 months

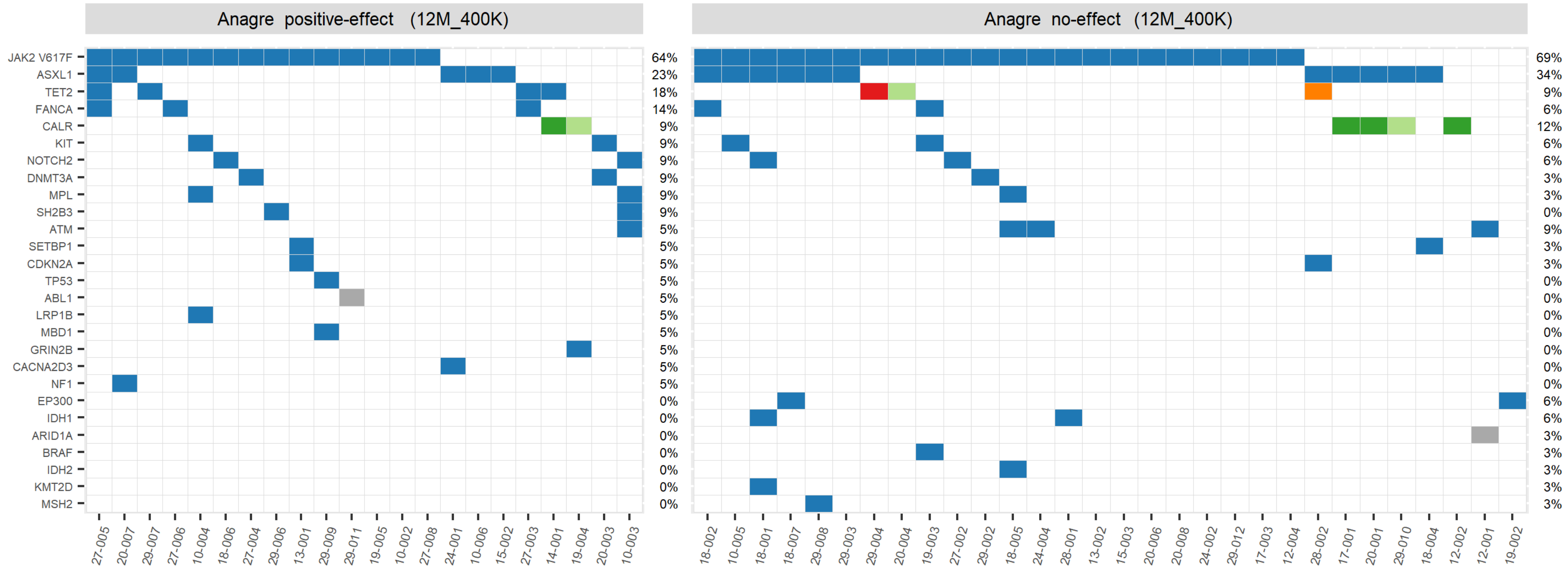

(D) Platelet dropping by 50% vs not at 12 months

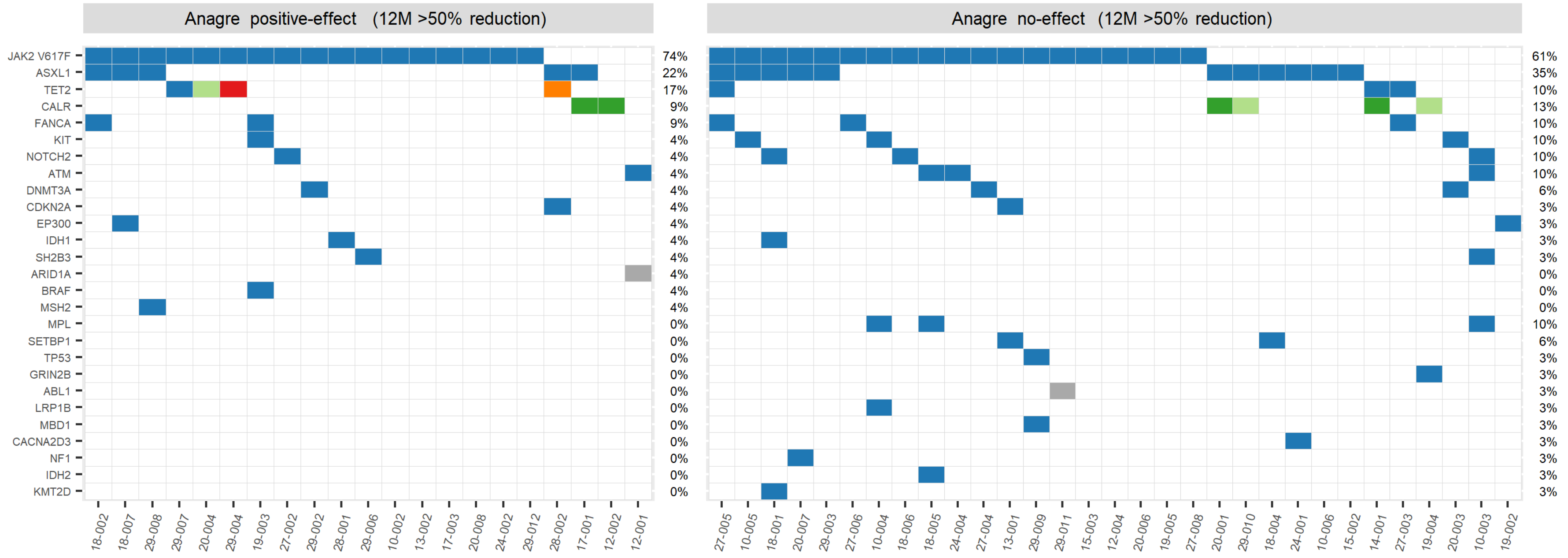

References

1. McKenna, A. *et al.* The Genome Analysis Toolkit: a MapReduce framework for analyzing next-generation DNA sequencing data. *Genome Res* **20**, 1297-1303, doi:10.1101/gr.107524.110 (2010).
2. Van der Auwera, G. A. *et al.* From FastQ Data to High-Confidence Variant Calls: The Genome Analysis Toolkit Best Practices Pipeline. *Current Protocols in Bioinformatics* **43**, 11.10.11-11.10.33, doi:https://doi.org/10.1002/0471250953.bi1110s43 (2013).
3. Bolger, A. M., Lohse, M. & Usadel, B. Trimmomatic: a flexible trimmer for Illumina sequence data. *Bioinformatics* **30**, 2114-2120, doi:10.1093/bioinformatics/btu170 (2014).
4. Poplin, R. *et al.* Scaling accurate genetic variant discovery to tens of thousands of samples. *bioRxiv*, 201178, doi:10.1101/201178 (2018).
5. Wang, K., Li, M. & Hakonarson, H. ANNOVAR: functional annotation of genetic variants from high-throughput sequencing data. *Nucleic Acids Res* **38**, e164-e164, doi:10.1093/nar/gkq603 (2010).
6. McLaren, W. *et al.* The Ensembl Variant Effect Predictor. *Genome Biology* **17**, 122, doi:10.1186/s13059-016-0974-4 (2016).
7. Karczewski, K. J. *et al.* The mutational constraint spectrum quantified from variation in 141,456 humans. *Nature* **581**, 434-443, doi:10.1038/s41586-020-2308-7 (2020).
8. Forbes, S. A. *et al.* COSMIC: somatic cancer genetics at high-resolution. *Nucleic Acids Res* **45**, D777-d783, doi:10.1093/nar/gkw1121 (2017).
